# Copper neurotoxicity: Induction of cognitive dysfunction: A review

**DOI:** 10.1097/MD.0000000000036375

**Published:** 2023-12-01

**Authors:** Duan Feng, Yu Zhao, Wei Li, Xuechao Li, Jixiang Wan, Fangjun Wang

**Affiliations:** a Department of Anesthesiology, Affiliated Hospital of North Sichuan Medical College, Nanchong, China; b General Surgery Department, Enyang District People’s Hospital, Bazhong City, China; c ICU, Bazhong District People’s Hospital, Bazhong, China.

**Keywords:** cognitive dysfunction, cognitive function, copper, copper exposure, copper homeostasis, cuproptosis

## Abstract

Cognitive dysfunction occurs mainly in certain diseases and in the pathological process of aging. In addition to this, it is also widespread in patients undergoing anesthesia, surgery, and cancer chemotherapy. Neuroinflammation, oxidative stress, mitochondrial dysfunction, impaired synaptic plasticity, and lack of neurotrophic support are involved in copper-induced cognitive dysfunction. In addition, recent studies have found that copper mediates cuproptosis and adversely affects cognitive function. Cuproptosis is a copper-dependent, lipoylated mitochondrial protein-driven, non-apoptotic mode of regulated cell death, which provides us with new avenues for identifying and treating related diseases. However, the exact mechanism by which cuproptosis induces cognitive decline is still unclear, and this has attracted the interest of many researchers. In this paper, we analyzed the pathological mechanisms and therapeutic targets of copper-associated cognitive decline, mainly in the context of neurodegenerative diseases, psychiatric and psychological disorders, and diabetes mellitus.

## 1. Introduction

Copper plays a key cofactor role in energy production, iron metabolism, antioxidants, connective tissue and neurotransmitter synthesis. Copper is also essential for cellular processes involved in cellular signaling, catecholamine homeostasis, neuronal myelin formation, and synaptic transmission in the central nervous system. Intracellular copper concentrations are kept relatively low, and increases can lead to cytotoxicity or cause cell death. Therefore, copper uptake, distribution, and elimination are tightly regulated, and copper enzymes, copper chaperones, and membrane transport proteins play a key role in copper homeostasis.

Excessive copper intake and dysregulation of copper homeostasis cause copper accumulation in the brain, and excess copper induces apoptosis or cuproptosis in neuronal cells, while neuronal loss leads to memory deficits and cognitive impairment.^[1]^ On the other hand, dysregulation of copper homeostasis induces the production of reactive oxygen species (ROS), which attack biological macromolecules, leading to oxidative damage to the proteome, lipids, and DNA, inducing apoptosis, exacerbating neuronal toxicity and dysfunction, and triggering cognitive impairment.^[2]^ Glutamate, malondialdehyde, and caspase-3 were elevated, and glutathione (GSH) was decreased in the frontal cortex and hippocampus of copper-exposed mice. At the same time, Y-maze attention scores were reduced. Suggesting that copper induces apoptosis in the hippocampus and frontal cortex via glutamate and oxidative stress pathways and leads to impaired memory and learning.^[3]^

Cuproptosis is a regulated cell death triggered by excess Cu^2+^ that is distinct from apoptosis. Copper binds to lipid-acylated components of the tricarboxylic acid (TCA) cycle, and the aggregation of these copper-bound lipid-acylated mitochondrial proteins and subsequent depletion of Fe-S cluster proteins induces proteotoxic stress and ultimately cell death.^[4]^ Recent studies have shown a dose-dependent increase in the copper-exposed mouse proteolipid acylase dihydrolipoamide S-acetyltransferase (DLAT), a downregulation of the Fe-S cluster protein Ferredoxin 1, DNA polymerase delta 1, and aconitase 2 expression, and an increase in the proteotoxic stress marker heat shock protein 70.^[5]^ Cuproptosis is recognized as a cause of cognitive dysfunction. As such, it offers new avenues for treating and understanding many diseases and pathological states.

## 2. Copper overload affects cognitive function

Data from the National Health and Nutrition Examination Survey showed an association between total copper intake and low cognitive performance on the Animal Fluency and Digit Symbol Substitution Test, which represents a negative association between copper intake and executive ability, processing speed, sustained attention, and working memory.^[6]^ A study that included 10,269 participants showed that greater dietary copper intake was associated with a decrease in overall language function.^[7]^ It has been shown that serum copper is negatively associated with working memory and executive function in healthy individuals and that serum copper concentrations are significantly increased in older adults with cognitive impairment.^[8,9]^ Analysis of data from National Health and Nutrition Examination Survey 2011 to 2014 showed that serum copper, although no association was found between the Digit Symbol Substitution Test and the Consortium to Establish a Registry for Alzheimer Disease (CERAD) Word Learning (CERAD-WL) and Delayed Recall (CERAD-DR) tests, was negatively associated with Animal Fluency scores, suggesting that serum copper is associated with executive function.^[10]^

The imbalance of copper homeostasis in the brain is associated with cognitive decline in several neurodegenerative diseases. In Alzheimer disease (AD), copper binds directly to Aβ and promotes Aβ accumulation and oligomerization, which exacerbates ROS production and leads to oxidative neuronal damage. In vitro, isolation of copper from Aβ prevents its accumulation and leads to Aβ degradation, inhibition of hydroxyl radical production and oxidative damage, and ultimately reduction of cell death.^[4]^ Several phase II trials have shown that the copper chelator clioquinol reduces Aβ aggregation and improves cognitive function. Its derivative PBT2 has also been shown to reduce Aβ levels and improve cognition in several Phase Ib/IIa trials.^[11]^ In Huntington disease (HD), copper accumulation promotes the aggregation of Huntingtin proteins and interacts with histidine residues at the N-terminal end of the protein.^[4]^ Copper may contribute to the pathogenesis of HD by inhibiting lactate dehydrogenase, a key enzyme involved in lactate metabolism.^[12]^ Copper chelators (clioquinol or tetrathiomolybdate) attenuate cognitive deficits and behavioral abnormalities in the R6/2 HD mouse model.^[13]^

Also, cognitive decline in some psychiatric disorders (schizophrenia, depression) has been associated with copper accumulation. A meta-analysis involving 39 studies showed that copper levels were higher in patients with schizophrenia compared to those without schizophrenia and higher in men than in women.^[14]^ According to a cross-sectional study, the prevalence of depression was significantly higher in samples with high copper levels.^[15]^ Recent studies have found that cognitive impairment and hippocampal atrophy, important features of type 2 diabetes, are significantly associated with elevated copper levels.^[16]^

## 3. Mechanisms by which copper overload affects cognitive function

### 3.1. Inflammation-related factors

#### 3.1.1. High mobility group box 1 (HMGB1).

HMGB1 is the most abundant non-histone nuclear protein that plays an important role in maintaining nucleosome structure and function, influencing gene transcription, DNA repair, and chromosome rearrangement. It can be secreted or released by stressed, dead, or dying cells. In aged rats, an increase in HMGB1 in the hippocampus is suspected to be associated with cognitive impairment. In aging patients, clinical studies have confirmed the threat of serum HMGB1 to cognitive function, and elevated levels of HMGB1 in peripheral blood correlate with postoperative cognitive dysfunction (POCD).^[17]^ HMGB1 induces alterations in endothelial cell function and increases blood-brain barrier (BBB) permeability, HMGB1 can interact with various receptors, including receptor for advanced glycation end-products (AGEs) (RAGE), and also with Toll-like receptor (TLR)-2 and TLR-4, which activate inositol-requiring transmembrane kinase/endoribonuclease 1alpha (IRE1α) in mouse macrophages.^[18,19]^ In AD, Cu accelerates AGE formation, and AGEs exacerbate neurotoxicity by upregulating RAGE expression and glycogen synthase kinase-3 activation. The interaction of AGEs with RAGEs on the surface of neuronal cells activates the nuclear factor-kappaB (NF-κB) pathway through Aβ-mediated oxidative stress.^[20]^

Copper accumulation appears to cause oxidative damage to mitochondrial membranes and decrease enzyme activity in the TCA cycle, as well as mitochondrial morphological abnormalities, including the reduction or elimination of mitochondrial cristae, resulting in ATP depletion. ATP depletion activates adenosine-activated protein kinase (AMPK), which enhances phosphorylation of HMGB1, resulting in greater extracellular release. In vitro, the AMPK inhibitor Dorsomorphin inhibited copper-induced cell death and HMGB1 release.^[21]^ This suggests that HMGB1 is a key immune mediator of cuproptosis-induced sterile inflammation.

#### 3.1.2. NLRP3 inflammasome.

The NLRP3 inflammasome consists of the innate immune receptor protein NLRP3, the adaptor protein ASC, and caspase-1.^[22]^ Activated caspase-1 cleaves gasdermin D (GSDMD), and the N-terminal structural domain of GSDMD forms a pore in the plasma membrane, which triggers a cleavage-promoting pro-inflammatory form of cell death called pyroptosis. Aberrant activation of the NLRP3 inflammasome is associated with the pathogenesis of several neurodegenerative diseases, such as AD and Parkinson disease. Animal experiments have shown that the NLRP3 initiation state in the brain of aged mice may be associated with isoflurane-induced hippocampal inflammation and cognitive impairment,^[23]^ and inhibition of NLRP3 signaling can improve POCD in aged mice.^[24]^ For depression, NLRP3 activation regulates caspase-1 activation, promotes maturation of IL-1β and IL-18 in microglia, and activates microglia.^[25]^

Nikolaus Deigendesch study showed that depletion of bioavailable copper by the copper chelator tetrathiomolybdate specifically inhibited macrophage caspase-1 activation as demonstrated in in vivo and in vitro experiments.^[26]^ Copper promotes pyroptosis by inducing ROS production and endoplasmic reticulum (ER) stress, leading to the formation of the NLRP3 inflammasome and generating membrane pores that cause cell death through the action of GSDMD.^[27]^ Excess copper promotes ROS formation through the Fenton reaction or by lowering GSH.^[28]^ Yanan An’s study showed that ROS are upstream signals for mitogen-activated protein kinases, NF-κB and the NLRP3 inflammasome. In addition, mitogen-activated protein kinases mediates the activation of NF-κB and NLRP3, whereas NF-κB upregulates NLRP3 inflammasome, and ROS can also dissociate thioredoxin-interacting protein (TXNIP) from thioredoxin and allow it to bind to NLRP3, leading to the activation of the NLRP3 inflammasome.^[18,29]^ It has been shown that inhibition of the NF-κB signaling pathway by JQ1 attenuates cognitive deficits induced by anesthesia (especially prolonged anesthesia).^[30]^

#### 3.1.3. cAMP response element-binding protein (CREB).

Immune system dysfunction and inflammation are associated with loss of blood CREB and altered CREB signaling in the brain, with adverse consequences such as cognitive decline. CREB is converted to the activated CREB (p-CREB) form by phosphorylation of Ser133, and CREB is involved in the transcription of genes related to long-term potentiation formation and synaptic plasticity, such as Egr-1, along with the transcriptional cofactor CREB binding protein. Also, in AD patients, p-CREB expression in peripheral blood mononuclear cells (PBMCs) correlates with expression in the brain and alters CREB signaling in the brain, thus exacerbating cognitive decline in AD.^[31]^ Disturbances in CREB phosphorylation and transcriptional mechanisms interacting with CREB play a key role in synaptic dysfunction and memory loss.^[32]^

It has been shown that copper blocks CREB phosphorylation, thereby reducing the expression of its downstream target protein, brain-derived neurotrophic factor (BDNF), leading to cognitive dysfunction in mice.^[5]^ BDNF regulates cell survival, differentiation, neurite outgrowth and regeneration, and synaptic plasticity in both the central and peripheral nervous systems.^[33]^ BDNF deficiency is strongly associated with age-related hippocampal dysfunction and may trigger memory impairment and increase the risk of depression. For schizophrenia, higher levels of BDNF have a permissive role in allowing SOD to improve cognition.

### 3.2. Proteins, neurotransmitters, receptors

#### 3.2.1. Claudin protein.

The integrity of the BBB depends on many factors, including a specific set of claudin proteins.^[34]^ claudins-1, and claudins-5 are major components in the formation of BBB endothelial tight junctions, and mice deficient in claudin-5 exhibit severely impaired and leaky BBB.^[35]^ Studies have shown that claudin-1, claudin-3, and claudin-5 protein expression levels all exhibit significant downregulation in a copper-dependent manner.^[36]^

The BBB keeps central and peripheral transmitter pools separate and protects the brain from changes in plasma glutamate levels. The BBB prevents the entry of many macromolecules into the brain via paracellular or diffusive pathways. For example, leakage of plasma proteins such as albumin, prothrombin, and fibrinogen has a detrimental impact on neural tissues, leading to neuroglia activation, glia division, and consequently, apoptosis. Damage to the BBB causes the infiltration of different plasma components and immune cells into the brain parenchyma and microglia activation, leading to central inflammation.^[37]^ The BBB is involved in the pathogenesis of AD, and its dysfunction triggers neuroinflammation and oxidative stress, promotes Aβ production, and leads to cognitive dysfunction.^[38]^ BBB disruption in elderly patients is associated with more rapid cognitive decline.^[39]^ Also, the cognitive decline associated with vascular dementia is associated with BBB function.^[40]^ A significant reduction of claudin-5 in brain tissue in a mouse model of POCD has been reported to exacerbate isoflurane-induced POCD.^[41]^

#### 3.2.2. X-box binding protein 1 (XBP1), IRE1α, PKR-like ER kinase (PERK).

In vitro and in vivo experiments showed that copper nanoparticles triggered oxidative stress and activated the ER stress pathway, leading to the opening of the apoptotic pathway of C/EBP homologous protein (CHOP), c-Jun N-terminal kinase, and Caspase-12.^[42]^ Copper also increased the expression of ER stress proteins such as XBP1, PERK, and CHOP.^[43]^ ER stress triggers an unfolded protein response with molecular signaling involving IRE1α and its downstream XBP1, activating transcription factor-6, and PERK.

IRE1α activation increased mitochondrial ROS levels and facilitated binding between NLRP3 and mitochondria, and IRE1α binding to factor (TNF) receptor associated factor-2 activated c-Jun N-terminal kinase-activator protein 1 and NF-κB, increasing IL-6 and TNF-α production. In addition, IRE1α activates retinoic acid-inducible gene I via regulated IRE1alpha-dependent decay and triggers inflammatory responses by activating NF-κB via mitochondrial antiviral signaling protein. PERK induces NF-κB in a nucleotide-binding oligomerization domain 1-dependent manner and PERK/ eukaryotic translation initiation factor 2 alpha (eIF2α)/ATF4, PERK phosphorylates eIF2α, which partially triggers downstream TXNIP and NF-κB activation, and the PERK-eIF2α-ATF3 signaling pathway promotes CHOP expression, which activates the NLRP3 inflammatory vesicle.IRE1α also activates NLRP3 inflammatory vesicles through unconventional XBP1 splicing and inhibition of MiR-17-5P.^[18]^ Unfolded protein response impairment is directly linked to neurodegeneration, and PERK and IRE1α-XBP1 signaling are associated with neurodegenerative diseases. Because they mediate the reduction of chronic endoplasmic reticulum stress and affect synaptic function.^[44,45]^ ER stress weakens endoplasmic reticulum function, leading to the accumulation of unfolded or misfolded proteins, causing apoptosis and neurodegeneration, which are associated with many neurodegenerative diseases, including AD and Parkinson disease.^[46]^ ER stress-induced downregulation of synaptic proteins leads to diminished intercellular signaling, causing cognitive impairment.^[47]^

#### 3.2.3. Neurotransmitter.

Copper exposure decreased the expression of synaptophysin (SYP), postsynaptic density protein-95 (PSD-95), and transmitters including dopamine, 5-HT, and GABA in mice.^[5]^ Synaptophysin and PSD-95 are jointly involved in the regulation of synaptic plasticity, and dopamine, 5-HT, and GABA are closely associated with synaptic strength and regulation. Dysregulation of these factors may affect learning and memory.^[48,49]^

Cu toxicity induces apoptosis and astrocytosis in the hippocampus and frontal cortex via the glutamatergic pathway, leading to impaired memory and learning.CuSO4-treated mice had elevated levels of glutamate in the hippocampus, and glutamate was increased in the frontal cortex at day 90.^[3]^ TNF-α induces an enhanced alpha-amino-3-hydroxy-5-methyl-4-isoxazolepropionic acid/N-methyl-d-aspartate pathway and indirectly inhibits the glutamate transporter on astrocytes by binding to CREB to induce glutamate transporter expression decreases, leading to glutamate toxicity. The role of glutamate as an excitatory neurotransmitter and its receptors in learning and memory is widely recognized. Glutamate is broken down into glutamine for reuse via postsynaptic receptors or astrocytes. In addition, excess glutamate in the synaptic gap causes activation of N-methyl-D-aspartate receptor and Alpha-amino-3-hydroxy-5-methyl-4-isoxazolepropionic acid receptors, leading to Ca entry into the cell, causing cytoskeletal and DNA damage, mitochondrial swelling, ROS production, and ultimately energy depletion and apoptosis.^[50]^ The N-methyl-D-aspartate receptor antagonist memantine ameliorated the lack of pleasure, anxiety, and cognitive deficits associated with depression in rats, which was associated with inhibition of serum copper ion levels.^[51]^ Dehydroglyasperin C, the active component of licorice root, inhibits activator protein 1 and NF-κB to reduce LPS-induced microglia activation and inflammation. Licorice-derived isoliquiritigenin inhibits glutamate-associated neurotoxicity by reducing stress mediators (ROS, membrane lipid peroxidation, calcium, and apoptotic factors). In addition, by inhibiting ROS formation and blocking the release of apoptotic factors (Bcl2, Bax, and AIF) from mitochondria into the cytosol.^[52]^

#### 3.2.4. Mitochondrial protein.

Studies have shown that downregulation of mitochondrial proteins such as GRP75 and GRP78 is involved in neurotoxicity induced by chronic low-dose copper exposure.^[53]^ Copper overload induces oxidative damage to mitochondrial membranes and impairs the function of enzymes in the TCA cycle. Copper exposure induces early inhibition of pyruvate dehydrogenase and α-ketoglutarate dehydrogenase activities and decreases the mitochondrial transmembrane potential (ΔΨm) in neuronal and hepatocytes.^[54]^ Decreased ΔΨm opens the permeability transition pore, increases membrane permeability, and leads to electron leakage in the respiratory chain complex and ultimately apoptosis. Exposure of neuroblastoma cells to copper significantly increased mitochondrial ROS (mtROS) production, caused mitochondrial DNA (mtDNA) damage, and decreased pyruvate dehydrogenase production in the TCA cycle and respiratory complex I.^[55]^ On the other hand, the NLRP3 inflammasome can be enhanced by mtROS and mtDNA; mtROS induces TXNIP binding to leucine repeat fragments of NLRP3, while mtDNA is released from damaged mitochondria into the cytoplasm through direct binding to NLRP3.^[17]^ Nakahira et al found that mtROS and mtDNA produced by dysfunctional mitochondria are required for NLRP3 inflammasome activation.^[23]^

In mitochondria, copper directly binds DLAT and promotes the disulfide-bond-dependent aggregation of lipoylated DLAT. Copper also mediates the disruption of iron-sulfur (Fe-S) enzymes, and copper prevents the formation of Fe-S clusters by inhibiting the activity of associated mitochondrial assembly proteins, such as the disruption of Fe-S clusters in mitochondrial Ferredoxin 1, which reduces protein lipid acylation and inhibits cuproptosis.^[4]^ Lipoylated protein aggregation and loss of Fe-S cluster proteins trigger proteotoxic stress and ATP depletion, which ultimately mediate cuproptosis.

### 3.3. Neuroglia

#### 3.3.1. Microglia.

During brain development, microglia regulate synaptic transmission, prune neuronal synapses, and facilitate the formation of neural circuits. Microglia can be polarized into “M1” or “M2” phenotypes depending on the brain microenvironment. M1 secretes pro-inflammatory molecules such as TNF-α, IFN-γ, IL-1β, IL-6, NO, and ROS,^[56]^ which inhibit normal neuronal growth and adversely affect synaptic transmission. Microglia are also involved in cell death, and in a mouse model of spinal cord injury, necrotic components are located not only in the cell membrane but also in the endoplasmic reticulum of microglia and macrophages, suggesting that microglia are involved in cell necrosis via endoplasmic reticulum stress. At the same time, endoplasmic reticulum stress activates the TXNIP signaling pathway and promotes apoptosis.^[17]^ In neurodegenerative diseases, abnormal aggregation of Aβ, synaptic nuclear protein, or superoxide dismutase 1 activates microglia and induces increased IL-1β release, leading to neurodegeneration and cognitive impairment.^[57]^

Animal experiments have shown that chronic copper exposure increases the risk of the degenerative transformation of microglia and leads to accelerated cognitive decline in AD mice.^[58]^ Also, 24-hour copper exposure inhibited phagocytosis or low-density lipoprotein receptor-related protein 1-dependent phagocytosis of mouse microglia, which attenuated Aβ clearance and caused increased release of pro-inflammatory cytokines such as IL-1β, TNF-α, and IL-6.^[28]^ Microglia activation may be associated with copper-induced oxidative stress and activation of the NF-κB pathway. It has been shown that Cu-binding peptide (Cu-bp) inhibits the translocation of NF-κB p65 to the nucleus to alleviate the inflammatory response of microglia.^[59]^ Accumulation of copper in cells leads to disrupted mitochondrial autophagy and overexpression of the NLRP3/caspase-1/GSDMD protein axis in microglia. Copper also activates the secretion of NO, TNF-α and IL-6 by M1-type microglia.^[60]^ Yi Huang’s study showed that TNF-α binding to TNFR leads to activation of NF-κB and subsequent initiation of NLRP3.^[22]^ Studies have shown that TNF-α signaling exacerbates Aβ deposition and tau protein formation in vivo and that effective TNF-α inhibitors (etanercept) have the potential to improve cognitive performance in AD patients.^[61,62]^

#### 3.3.2. Astrocyte.

Astrocytes play a key role in neuronal circuits that control emotion, learning, and memory,^[63]^ and their essential functions include regulation of copper homeostasis, metabolic supply of neurons, maintenance of the BBB, and regulation of synaptic transmission and synaptic plasticity.^[64]^ Astrocytes release various soluble factors, such as glial cell line-derived neurotrophic factor, transforming growth factor-β, basic fibroblast growth factor, and angiopoietin-1, which regulate angiogenic processes and the formation of endothelial cell junctions, thereby maintaining the structural and functional integrity of the BBB. Astrocytes can also use mechano-gated Piezo1 channel-mediated mechanotransduction mechanisms to robustly regulate adult neurogenesis and cognitive function.^[65]^ In AD, Aβ stimulates NF-κB and complement signaling in astrocytes, inducing the synthesis of inflammatory mediators (IL-1, C1q, and TNF-α) in astrocytes, impairing synaptic density and dendritic morphology.^[66]^

When astrocytes are exposed to copper for a prolonged period of time, the copper storage capacity of the cells increases (with increased levels of GSH and MTs).^[67]^ Their significant resistance to copper-induced toxicity has been reported,^[68]^ which is overwhelmed when copper uptake is very rapid.^[69]^ In in vivo studies, excess Cu decreases cell viability of cultured astrocytes, and subcytotoxic concentrations of Cu impair ΔΨm and affect mitochondrial function, ultimately triggering ROS production and leading to hypertrophy or death of human astrocytes.^[70]^ Cu in the neocuproine complex may cause apoptosis in rat cortical astrocytes by inducing ROS production, a decrease in ΔΨm, and depletion of GSH and ATP.^[68]^ In AD, excess non-copper blue protein Cu triggers reactive astrocyte proliferation, which may lead to an irreversible neuroinflammatory response. Interaction between Aβ oligomers and astrocyte TLR 2/4 can stimulate an inflammatory response, which triggers toxic pro-inflammatory mediators affecting the neurons, including IL-1β, IL-6, macrophage inflammatory protein (MIP), and NO.^[71]^

### 3.4. Others

#### 3.4.1. Ferroptosis.

Iron is essential for normal neuronal function; excess iron in the brain is associated with several neurodegenerative diseases; and disturbances in iron metabolism may be linked to the pathogenesis of general anesthesia-induced neurotoxicity and cognitive deficits, which may be induced through ferroptosis.^[72]^ Copper enhances the biosynthesis of circulating iron oxide ceruloplasmin, and ceruloplasmin enhances iron release from storage. Also, copper affects the DNA-binding activity of hypoxia-inducible factor and the activity of hepcidin, thereby regulating iron homeostasis.^[73]^ It was shown that copper promotes ferroptosis by inducing ROS production and macroautophagy/autophagic degradation of glutathione peroxidase 4.^[74,75]^

#### 3.4.2. Microbiota-gut-brain axis.

Gut microbiota information can be transmitted to the central nervous system via the autonomic nervous system and vagus nerve.^[76]^ The gut microbiota can regulate the transcription of genes such as PGC-1α, sirtuin 1, and AMPK in mitochondria and affect mitochondrial energy metabolism, ROS production, and inflammatory responses. Changes in gut microbiota disrupt the intestinal mucosal barrier and BBB, triggering inflammation, oxidative stress, and mitochondrial dysfunction. Higher BBB permeability in mice lacking physiologic gut microbiota is associated with reduced expression of tight junction proteins (occludin and claudin-5). Increased intestinal permeability and disruption of the central nervous system barrier provide a gateway for intestinal lumen-derived molecules, toxins, and pathogens to reach the brain parenchyma, activate local immune cells, and trigger neuroinflammation.^[77]^ Gut flora can influence brain function by modulating the transmission of serotonergic, noradrenergic, dopaminergic, glutamatergic, and GABAergic neurotransmitters, and the microbiota can influence the synthesis or metabolism of neurotransmitters as well as produce these neuroactive substances on their own.^[78]^ Gut microbiota imbalance is suspected to have neurological effects on the host and to increase the risk of POCD and neuropsychiatric disorders.^[79,80]^ Exposure to copper in mammals resulted in alterations in the composition, abundance, and diversity of the gut microbiota^[81,82]^ and was accompanied by structural abnormalities in the intestinal epithelium, with marked vacuolization and enlargement of the lamina propria and nuclear shrinkage.^[83]^

## 4. Conclusion

In summary, copper overload plays an important role in the mechanism of cognitive impairment. Central Aβ aggregation, oxidative stress, and neuronal apoptosis have been shown to have a definite link with copper-induced cognitive impairment. In this review, the mechanisms by which copper affects cognitive impairment also involve inflammatory factors, neurotransmitters and receptors, neuroglia, and copper-associated cell death. In addition, some copper chelators may have beneficial effects on cognitive function. For example, clioquinol and PBT2 reduce Aβ aggregation and improve cognitive function. Tetrathiomolybdate inhibits pyroptosis and improves cognitive function by inhibiting NLRP3 inflammasome activation. In addition, the NF-κB inhibitor JQ1 and the plant licorice root have shown some advantages in attenuating neurotoxicity. However, these drugs require further in vivo and clinical studies to infer their methods of action. Future research should prioritize well-designed human clinical trials to explore the specific mechanisms of copper toxicity, gain a comprehensive understanding of the involvement of glial cells, elucidate detailed molecular pathways, and further investigate the microbiota-gut-brain axis. Meanwhile, the mechanisms by which cuproptosis affects cognitive function are unclear, and the mechanisms need to be further explored.

## Acknowledgments

The authors would like to thank all the authors and institutions involved in this study for their contributions to this paper.

## Author contributions

**Formal analysis:** Xuechao Li.

**Investigation:** Duan Feng.

**Methodology:** Yu Zhao, Wei Li.

**Resources:** Wei Li.

**Supervision:** Xuechao Li, Jixiang Wan.

**Validation:** Jixiang Wan, Fangjun Wang.

**Writing – original draft:** Duan Feng.

**Writing – review & editing:** Fangjun Wang.
